# Grand Challenges for Biosafety and Biosecurity

**DOI:** 10.3389/fbioe.2014.00035

**Published:** 2014-09-17

**Authors:** Kenneth I. Berns

**Affiliations:** ^1^Department of Molecular Genetics and Microbiology, University of Florida College of Medicine, Gainesville, FL, USA

**Keywords:** biosafety, biosecurity, grand challenges, dual use research of concern

Success in the prevention and treatment of infectious diseases has saved more lives than any other achievement of medical science. The three greatest advances have been clean water, immunization, and the advent of antibiotics and, more recently, antivirals. Despite this record of success, we are far from conquering the grand challenge to human health posed by microbes. All that has been achieved is the consequence of increasingly sophisticated research. Through advances in technology that have led to deep sequencing, we are now able to identify previously unrecognized bacteria and other microbes, which had previously eluded our appreciation because of our inability to culture them. One of the consequences has been an increased understanding of the omnipresence of bacterial populations in the human body. It is now estimated that the human body on average contains 10 times as many bacterial cells as human cells (Savage, 1977; Berg, 1996). In addition, the human body probably contains at least 10 times as many viral genomes as bacterial genomes (Lower et al., 1996; Downey et al., 2014). Thus, humans are very much a diverse biological community and it behooves us to gain as complete knowledge as possible if we are to continue to enhance human health.

Because much of the research in this area involves study of microbial pathogens, there is potential risk of infection for researchers in the laboratory. Additionally, there is the risk that dangerous microbes under study might escape from the laboratory and pose a risk to the public health (as has been voiced in the case of highly pathogenic avian influenza H5N1). Although many of the microbes under study are normally present in the environment, in special cases the bacteria or viruses have natural or enhanced virulence not usually present in the environment and these are of especial concern. Pathogens have been classified with respect to the degree of risk they represent, both to lab workers and to the public. The assessment depends not only on the virulence of the microorganism, but also on whether an effective vaccine exists and/or effective antimicrobial/antiviral therapy is available. Where an effective vaccine exists, laboratory workers should be immunized and precautions should be taken to physically contain the microbes under study. Depending on the assessed virulence, various levels of physical containment have been mandated. In the United States, there are functionally four levels. Level one assumes no measurable risk and level four is for the most virulent pathogens for which no effective immunization or antimicrobial therapy exists. The U.S. Centers for Disease Control and Prevention (CDC) and the NIH publish a manual of recommendations for the physical containment of pathogens entitled “Biosafety in Microbiological and Biomedical Laboratories” (Abraham et al., 2009). These recommendations have been incorporated into regulations when the bacteria and viruses under study have been designated as select agents [see National Select Agent Registry (2014) for criteria used], which possibly could be used for biological warfare or bioterrorism.

Despite the efforts to promote biosafety, biosecurity, and good practices, assuring adherence to the prescribed practices remains a serious challenge. Although accidental laboratory acquired infections are rare and despite the fact that there have been no proven disease outbreaks attributable to release (“escape”) of pathogens from a laboratory, laboratory accidents and incidents do happen with a measurable frequency (Henkel et al., 2012). [It should be acknowledged that there is a strong suspicion that the 1977 H1N1 influenza outbreak was the consequence of a laboratory release (Webster et al., 1992).] There have been reports of three serious incidents in the U.S. in the past several weeks (Frieden, 2014b). A high containment laboratory at the CDC attempted to inactivate *Bacillus anthracis* using a non-validated method and then transferred the sample to a lower containment facility for further study. A week later, it was discovered that the sample had not been completely inactivated, which resulted in about 80 workers being potentially exposed. These workers were all offered immunization and antibiotic prophylaxis. Three laboratories had to be shut down and decontaminated. Fortunately, no one became ill. Secondly, a laboratory at the CDC transferred a culture of a non-virulent (for humans) strain of avian influenza to a U.S. Department of Agriculture laboratory where it later was discovered that the culture was contaminated with highly virulent H5N1 avian influenza virus. Finally, as a unit of the U.S. Food and Drug Administration (FDA) housed at the National Institutes of Health (NIH) prepared to move to a new campus, a carton in a cold room was discovered to contain six sealed vials of smallpox (Guillemin, 2014), a virus only permitted by treaty to be present at the CDC and one site in Russia. Apparently, the vials had been in the cold room for almost 50 years, because no one had ever bothered to either clean out or inventory the space. According to the CDC, the smallpox, which had been lyophilized, was still viable (Frieden, 2014a). Additional vials containing yet different pathogens were present in the same carton. These three incidents are all too graphic examples of the consequences of inadequate biosafety and biosecurity and point to the necessity for increased attention to these challenges.

Use of microbes as a weapon has a long history; examples include catapulting dead bodies into besieged cities in ancient times and giving blankets previously used for smallpox patients to Native Americans during the colonial period. State sponsored research in biological warfare continued through World War II until the biological weapons convention was signed in 1972. Despite having signed the convention, the Former Soviet Union apparently continued development and production of offensive biological weapons until 1990 (Weiner, 1998). One consequence of this activity was the accidental aerosol release of *B. anthracis* spores from a production facility in the Soviet city of Sverdlovsk in 1979, which resulted in multiple deaths (Meselson et al., 1994). Post 1990, we do not have irrefutable evidence of state sponsored research for the development and/or production of biological weapons, although research into defensive measures continues. Today, we are most concerned by the possible use of biological weapons by terrorists. There have been multiple instances of this on a small scale. In the United States, the best known example of this was mailing of letters containing *B. anthracis* spores to public officials and members of the media. Although only five people died as a result, this terrorist act cost over 500 million dollars for remediation of contaminated facilities and led to the introduction of significant legislation to regulate research involving select agents, i.e., those agents thought likely to be used by terrorists (Biosecurity, 2012). As the anthrax incident graphically demonstrated, a relatively small attack can have an enormous public (and economic) impact (Gursky et al., 2003).

With the advent of modern molecular genetics and rapid DNA sequencing, and the development of genetic engineering, the ability to analyze the molecular mechanisms underlying microbial pathogenicity has been enormously enhanced as has the ability to engineer desired properties into bacteria or viruses. Much of this work can be done in relatively modest facilities, which raises the concern that would be bioterrorists have the potential to develop their own bioweapons.

Especially after the anthrax incident of 2001, the concept of dual use research received much more attention in the United States (Biotechnology Research in an Age of Terrorism, 2004). In fact, it is possible to perceive of both good and bad uses of almost any research results. Experiments of most concern are those with results that might be directly misapplied. The US National Science Advisory Board for Biosecurity (NSABB) has defined seven types of experiments that fall into this area, which are designated dual use research of concern or DURC (DURC). “These are research that might (1) enhance the harmful consequences of a biological agent or toxin, e.g., information on how to make a seasonal strain of influenza as deadly as the 1918 pandemic virus; (2) disrupt immunity or the effectiveness of an immunization without clinical or agricultural justification, e.g., insertion of an immunosuppressive cytokine into a viral genome to render the antiviral immune response less effective; (3) confer to a biological agent resistance to a clinically and/or agriculturally useful prophylactic or therapeutic intervention against that agent or facilitate their ability to evade detection methodologies, e.g., information on how to confer doxycycline resistance to *Vibrio vulnificus*; (4) increase the stability, transmissibility, or the ability to disseminate a biological agent or toxin, e.g., information on changing genetic factors to increase transmissibility; (5) alter the host range or tropism of a biological agent or toxin, e.g., knowledge of how to convert non-zoonotic agents into zoonotic agents; (6) enhance the susceptibility of a host population, e.g., information on how to create a stable recombinant *Lactobacillus caseii* that could effectively block the host’s ability to synthesize an important immune signal, such as tumor necrosis factor alpha, which may directly facilitate the evasion of normal host defenses; and (7) generate a novel pathogenic agent or toxin or reconstitute an eradicated or extinct biological agent, e.g., information on how to construct a *de novo* microbial pathogen using unique gene sequences that do not exist in nature, or how to reconstitute a pathogen that no longer exists in nature, such as the 1918 pandemic influenza virus.”

Consideration for ways to deal with DURC are several. The first one is who determines that a research project meets the definition of DURC. The primary consideration should be by the investigator and the local institution. Equivalent responsibility should rest with the funding agency. However, experience has shown that research is being done and reported that meets the DURC definition. Often the journal to which the manuscript is submitted is the final filter. On several occasions, the editor has prevailed upon the authors to redact or modify certain details of the research. In one well reported case, two papers reporting gain of function experiments with avian influenza H5N1 were brought to the NSABB for review (Herfst et al., 2012; Imai et al., 2012). Initially, the NSABB recommended that certain details of both papers be redacted and only be made available to individuals with a certifiable need to know (Berns et al., 2012a,b). After modification of both manuscripts and additional consideration by NSABB, the modified manuscripts were recommended for publication. A major problem raised by the initial recommendation was the question of who could make the decision about who should be able to receive the full information. Because the site of the research in one instance was in the Netherlands and in another instance the journal involved was international, an authority able and willing to decide on distribution did not exist. Under current conditions, there is nothing to prevent an author whose work is considered by one journal to be DURC, from shopping the manuscript around until he/or she finds one that is willing to publish the manuscript without modification. As a consequence of the H5N1 experience, the US government established a policy to make the funding agency decide upfront about the question of DURC [see DURC Determination (2014)]. However, this does not resolve questions arising from the international nature of science. Further consideration of the issue at the international level is urgently required.

Both biosafety and biosecurity raise fundamental questions about the conduct of science, which deserve the attention of the scientific community.

## Conflict of Interest Statement

The author declares that the research was conducted in the absence of any commercial or financial relationships that could be construed as a potential conflict of interest.

## References

[B1] Abraham D. Adams L. G. Adler M. Alderman L. Ansell C. E. Barringer A. (2009). Biosafety in Microbiological and Biomedical Laboratories. Atlanta, GA: Centers for Disease Control and Prevention

[B2] Berg R. (1996). The indigenous gastrointestinal microflora. Trends Microbiol. 4, 430–435 10.1016/0966-842X(96)10057-3 8950812

[B3] Berns K. I. Casadevall A. Cohen M. L. Ehrlich S. A. Enquist L. W. Fitch J. P. (2012a). Policy: adaptations of avian flu virus are a cause for concern. Nature 482, 153–154 10.1038/482153a 22294204

[B4] Berns K. I. Casadevall A. Cohen M. L. Ehrlich S. A. Enquist L. W. Fitch J. P. (2012b). Policy: adaptations of avian flu virus are a cause for concern. Science 335, 660–661 10.1126/science.1217994 22294736

[B5] Biosecurity. (2012). Available at: http://osp.od.nih.gov/office-biotechnology-activities/biosecurity/dual

[B6] Biotechnology Research in an Age of Terrorism. (2004). National Research Council. The National Academies Press Available at: http://www.nap.edu/openbook.php?isbn=0309089778 25057686

[B7] Downey R. F. Sullivan F. J. Wang-Johanning F. Ambs S. Gile F. J. Glynn S. A. (2014). Human endogenous retrovirus K and cancer: innocent bystander or tumorigenic accomplice. Int. J. Cancer10.1002/ijc.29003 24890612 PMC6264888

[B8] DURC Determination. (2014). Available at: http://oba.od.nih.gov/biosecurity/biousgactivities.html

[B9] Frieden T. (2014a). Press Conference 07/11/2014 Available at: www.cdc.gov/media/releases/2014/t0711-lab-safety.html

[B10] Frieden T. (2014b). Report on the Potential Exposure to Anthrax. Centers for Disease Control and Prevention Available at: http://www.cdc.gov/about/pdf/lab-safety/Final_Anthrax_Report.pdf

[B11] Guillemin J. (2014). Smallpox: the long goodbye. Bull. Atom. Sci.

[B12] Gursky E. Inglesby T. V. O’Toole T. (2003). Anthrax 2001: observations on the medical and public health response. Biosecur. Bioterror. 1, 97–110 10.1089/153871303766275763 15040187

[B13] Henkel R. D. Miller T. Weyant R. S. (2012). Monitoring select agent theft, loss, and release reports in the United States-2004-2010. Appl. Biosafety 17, 171–180

[B14] Herfst S. Schrauwen E. J. Linster M. Chutinimitkul S. de Wit E. Munster V. J. (2012). Airborne transmission of influenza A/H5N1 virus between ferrets. Science 336, 1534–1541 10.1126/science.1213362 22723413 PMC4810786

[B15] Imai M. Watanabe T. Hatta M. Das S. C. Ozawa M. Shinya K. (2012). Experimental adaptation of an influenza H5 HA confers respiratory droplet transmission to a reassortant H5 HA/H1N1 virus in ferrets. Nature 486, 420–428 10.1038/nature10831 22722205 PMC3388103

[B16] Lower R. Lower J. Kurth R. (1996). The viruses in all of us: characteristics and biological significance of human endogenous retrovirus sequences. Proc. Natl. Acad. Sci. U.S.A. 93, 5177–5184 10.1073/pnas.93.11.5177 8643549 PMC39218

[B17] Meselson M. Guillemin M. Hugh-Jones A. Langmuir A. Popova I. Shelokov A. (1994). The Sverdlovsk anthrax outbreak of 1979. Science 266, 1202–1208 10.1126/science.7973702 7973702

[B18] National Select Agent Registry. (2014). Available at: http://www.selectagents.gov/

[B19] Savage D. C. (1977). Microbial ecology of the gastrointestinal tract. Annu. Rev. Microbiol. 31, 67–133 10.1146/annurev.mi31.100177.000543 334036

[B20] Webster R. G. Bean W. J. Gorman O. T. Chambers T. M. Kawaoka Y. (1992). Evolution and ecology of influenza viruses. Microbiol. Rev. 56, 152–179 1579108 10.1128/mr.56.1.152-179.1992 PMC372859

[B21] Weiner T. (1998). Soviet Defector Warns of Biological Weapons. The New York Times Available at: http://www.nytimes.com/1998/02/25/world/soviet-defector-warns-of-biological-weapons.html

